# The experience of patient partners in research: a qualitative systematic review and thematic synthesis

**DOI:** 10.1186/s40900-022-00388-0

**Published:** 2022-10-03

**Authors:** Jonathan Lauzon-Schnittka, Sophie Audette-Chapdelaine, Denis Boutin, Catherine Wilhelmy, Anne-Marie Auger, Magaly Brodeur

**Affiliations:** 1 Faculté de Médecine et des Sciences de la Santé, Université de Sherbrooke, Sherbrooke, QC Canada; 2 Département de Médecine Familiale et de Médecine d’urgence, Université de Sherbrooke, Sherbrooke, QC Canada; 3 Comité Stratégique Patient-Partenaire, Centre de Recherche du CHUS, Université de Sherbrooke, Sherbrooke, QC Canada; 4 Centre de Recherche du CHUS, Université de Sherbrooke, Sherbrooke, QC Canada

**Keywords:** Patient and public involvement, Patient engagement, Experience, Qualitative research, Systematic review

## Abstract

**Context:**

Patient engagement in research consists in involving patients as partners across the research cycle. This practice has quickly become an international standard, with funding bodies actively encouraging it. As the increased incentive to engage patients can lead to tokenistic partnerships, it is important to consider the experiences of patient-partners.

**Objective:**

To synthesize the qualitative literature on the experience of patients as partners in research.

**Design:**

A systematic review of the literature with thematic synthesis was realized, guided by the framework developed by Thomas and Harden (Bmc Med Res Methodol 8: 45, 2008).

**Data collection:**

A search strategy was developed to encompass keywords relating to patient-partners in research, their experience, and the qualitative nature of the target studies. 10 databases were searched using the EBSCO-host engine, along with the Scopus engine to include EMBASE. The search results were screened for the following inclusion criteria: articles written in English; articles reporting on the experience of patient-partners in research; qualitative studies or mixed-methods studies with a distinct qualitative section.

**Analysis:**

Included articles were charted for general information. The CASP qualitative checklist was used for critical appraisal. The “results” section of each article was coded line by line. Codes were aggregated inductively to form descriptive themes and analytical themes, in order to synthesize the ideas found in the selection of articles.

**Results:**

The initial search yielded 10,222 results. After the removal of duplicates, 5534 titles and abstracts were screened, 88 full-text reports were evaluated, and 41 studies were included. Articles reporting on these studies were published between 2005 and 2020. Seven themes emerged from the analysis: “motivations to engage in research”, “activities in patient engagement”, “structure”, “competence”, “team dynamics”, “impacts on broader life”, and “illness”. Articles reported varying degrees of perceived impact on research and satisfaction concerning the level of engagement. The importance of power differentials and team dynamics were widely stated.

**Conclusions:**

Findings provide an in-depth view of the experiences of patient-partners in research. Most articles reported a generally positive experience, but challenges and pitfalls of patient engagement were identified. This will serve research teams by highlighting good practices and possible improvements.

**Supplementary Information:**

The online version contains supplementary material available at 10.1186/s40900-022-00388-0.

## Plain English summary

Patient engagement is the practice of involving patients as partners in research teams. Through these partnerships, patients become co-researchers, which promotes patient-centered research. In recent years, this practice has quickly gained popularity and is now encouraged by scientific funding bodies. This financial incentive to engage patients helps to promote the practice but can lead to tokenistic partnerships. Therefore, it is important to understand how patient-partners experience engagement in research.

This systematic review aimed to describe the experience of patient-partners in research by synthesizing existing studies on the subject. 41 relevant studies were found, which described seven general themes in patient-partners’ experience. These themes were “motivations to engage in research”, “activities in patient engagement”, “structure”, “competence”, “team dynamics”, “impacts on broader life”, and “illness”. Within these themes, many positive experiences were described, such as the stimulating intellectual challenges of research, or the pride of helping others. Negative experiences were also identified, such as when patient-partners felt inadequately prepared for research activities, or when hierarchies of powers were formed. These findings will allow future research teams to improve patient engagement and will hopefully lead to better experiences for patient-partners.

**Supplementary Information:**

The online version contains supplementary material available at 10.1186/s40900-022-00388-0.

## Background

Patient engagement in research is the practice of involving patient-partners in the co-realization of health research across the research cycle [1–3]. In this context, the word “patient” refers to any person with past or present experience of health issues, personally or as a caregiver [1]. Patient engagement allows patients to become partners with academic researchers to create a “meaningful and active collaboration in governance, priority setting, conducting research and knowledge translation”, rather than participating only as research subjects [1]. Other terms are often used to refer to what this article will call “patient engagement”, such as the expression “patient and public involvement” that is more common in the United Kingdom. As this study was conducted in a Canadian institution, this article will use the expression “patient engagement” and considers it equivalent to its international synonyms.

It is argued that patients have the right to be involved in health research as they are the very people who will ultimately be affected by it [4]. Under this premise, patient engagement allows for the democratization of health research, by ensuring that the patient’s voice is heard, and therefore maintains patients at the center of health research [3, 4]. Furthermore, it is generally accepted that patient engagement can increase the quality of research [1, 4–6]. Indeed, patient-partners are experts in their own right: they have experiential, cultural, and circumstantial knowledge that academic researchers often lack. By bringing this different perspective to projects, patient-partners improve the relevance of research, its accessibility, and its uptake into healthcare [1, 4–6].

There exists substantial literature evaluating the impact of patient engagement on the research process. In a systematic review of 66 studies on this subject, Brett et al*.* [7] found that patient-partners improved research by, amongst other things: identifying patient-relevant research questions; adapting study protocols, questionnaires, and information sheets to be more appropriate for participants; providing greater access to the community and to participants; contributing to deeper insights from interviews and data analysis; and allowing broader dissemination of results. Regarding clinical trials, Crocker et al. [8] found that patient engagement strategies significantly increased the odds of participant enrolment in studies, particularly when the patient-partners had experienced the medical condition being studied. In an analysis of 126 reports of studies funded by the Patient-Centered Outcomes Research Institute (PCORI), Forsythe et al. [9] proposed 4 themes to describe the impacts of patient engagement in research: patient-partners increase the *acceptability* of studies by adapting protocols to reduce the burden on participants; patient-partners ensure the *feasibility* of studies by taking into account real-world obstacles; patient-partners contribute to the *rigor* of studies by enhancing data quality; patient-partners increase the *relevance* of studies by making research more patient-centered and by enhancing the dissemination of results.

Considering these arguments for patient engagement in research, it is easy to see why this practice has increased in popularity in the last few years. Indeed, patient engagement is becoming an international standard for health research, with funding bodies actively encouraging this practice [4, 10, 11]. However, this increased pressure to include patients in research can lead to tokenistic patient engagement, with researchers simply trying to “tick the right boxes” [12]. To ensure meaningful engagement, it is important to not only consider “*how much*” patient engagement is included but also “*how, why,* and *when*” [12]. A large portion of the answer to these questions lies in the experiences of patient-partners, as they are the cornerstone of patient engagement. Understanding their point of view is crucial for the development and promotion of this practice.

This study aimed to synthesize the qualitative evidence on patient research partners’ experience regarding their involvement in research. Specifically, this review aimed to qualitatively describe how patient engagement unfolds as seen and felt by patient-partners. It is hoped that by identifying strengths and weaknesses in current patient-engagement, this article will contribute to improving current practices.

## Methods

### Study design

A systematic review of the literature about the experience of patient research partners was conducted. The methodological framework developed by Thomas and Harden [13] guided the thematic synthesis of the data. The reporting of this study was guided by the *Enhancing transparency in reporting the synthesis of qualitative research (ENTREQ) statement* [14] (Additional File 1).

### Data collection

**Fig. 1:**
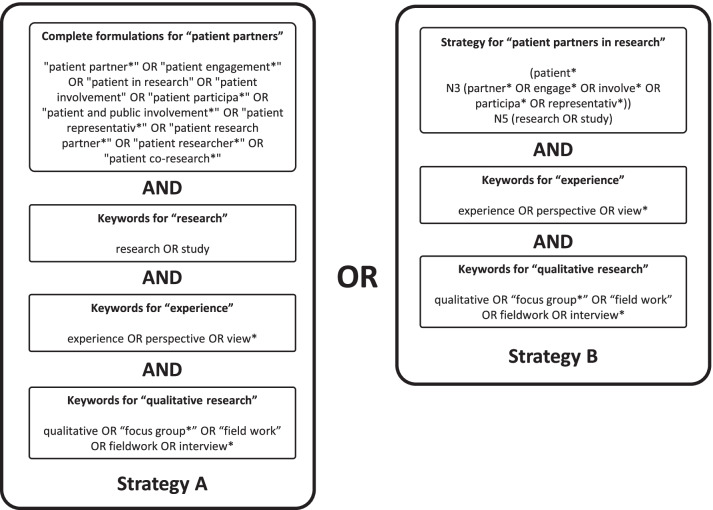
Summary of the search strategy

A comprehensive electronic search strategy was developed in collaboration with an information expert to include keywords relating to patient-partners in research, their experience, and the qualitative nature of the target studies. A sensible yet specific search strategy was developed to overcome two main barriers: the key concepts were very general, such as “research” and “experience”, and there is no standardized vocabulary used in the literature to describe “patient-partners”. To address these issues, two search strategies were combined (Fig. 1) to optimize the sensibility of the search, while keeping the number of results manageable. The strategy was adapted to each database searched with the use of controlled vocabulary (e.g. MeSH terms for MEDLINE and CINAHL Plus with Full Text). After consultation with an information expert, 10 databases were searched via EBSCO: MEDLINE with Full Text, Academic Search Complete, AgeLine, CINAHL Plus with Full Text, APA PsycInfo, APA PsycArticles, APA PsycExtra, Psychology and Behavioral Sciences Collection, Social Works Abstracts, SocINDEX. Scopus was also searched to include EMBASE, but MEDLINE was excluded from this search because it was already included in EBSCO. No other search limits or filters were applied. The search results were retrieved from the databases on June 28th, 2021. The complete search strategy used for each database can be found in the Additional File 2.

Eligibility of studies was determined using the following inclusion criteria: articles in which one of the main objectives was to study the experience of patient-partners in research; articles using qualitative methods; articles written in English. To encompass a broad range of perspectives, articles studying specific populations of patient-partners were included. To maximize the amount of useful data, mixed-method studies were included if they had a qualitative section that could be analyzed on its own. Similarly, studies that described both the experiences of patient-partners and those of academic researchers were included if it was possible to analyze only the views of the patient-partners.

### Study selection

Articles retrieved from the literature search were imported into the reference management software *ReadCube Papers*. After duplicates were removed, one author (JLS) screened titles and abstracts to determine eligibility for full-text review. Full-text reviews were conducted by the same author to determine the inclusion or exclusion of articles in this study. When there was uncertainty concerning the eligibility of articles, a team of authors (SAC, DB, CW, MB) was consulted to decide via consensus if the article was to be included or not.

### Data extraction and analysis

Data extraction and analysis were completed by JLS, and validated by SAC, DB, CW, and MB. General study characteristics were extracted. These included bibliographic information, aims, study design, participant information, and context of patient engagement. All selected articles were imported into the NVivo software, where the Results/Findings section of each article was coded line-by-line [13]. The other sections of the articles (e.g. Background and Discussion) served to better understand each study but were not used in the line-by-line analysis. For mixed-method studies, only the qualitative parts of the Results/Findings were extracted. For studies that described both the experiences of patient-partners and those of academic researchers, only the parts which concerned the patient-partners views were extracted. The line-by-line coding and identification of themes followed the methods for thematic synthesis described by Thomas and Haden [13]. Throughout the coding process, codes were inductively created when new ideas were identified, and ideas which had been previously seen were entered into the relevant codes. After the line-by-line coding was completed, codes which related to each other were grouped into descriptive themes and subthemes [13]. These were then aggregated into analytical themes that aimed to synthesize the main ideas found in the selection of articles. Once all articles were coded and themes were identified, all coded data were verified and recoded if necessary to maximize data representativeness. Codes were initially validated by SAC, and then by DB, CW, MB. To increase the depth of the analysis, counterexamples to already existing codes were explicitly sought.

### Quality assessment

All selected articles were evaluated for quality using the Critical Appraisal Skills Program qualitative checklist, as it is commonly used and applies to a wide range of qualitative designs [15]. Quality assessment was conducted by JLS and was verified by SAC and MB. Critical appraisal served to evaluate and comment on the credibility of findings. Studies were not excluded based on this evaluation, as there is currently no accepted method to do so in qualitative reviews [16, 17].

### Patient engagement and context

This review is part of a larger project that aims to describe the experiences of patient-partners in the research center at University of Sherbrooke. This larger project has been approved by the CIUSSS de l’Estrie—CHUS scientific committee and ethics committee and was initiated by two patient-partners (DB, CW) who currently sit on the Patient-Partner Strategic Committee of the research center. This committee has the mandate to promote “fruitful and long-lasting collaboration between patients and researchers”[18]. DB and CW co-authored this review, providing input on the selection of articles, on the codes and themes, and on the writing of this report. The protocol for this specific review has not been registered.

## Results

### Search and study selection

**Fig. 2:**
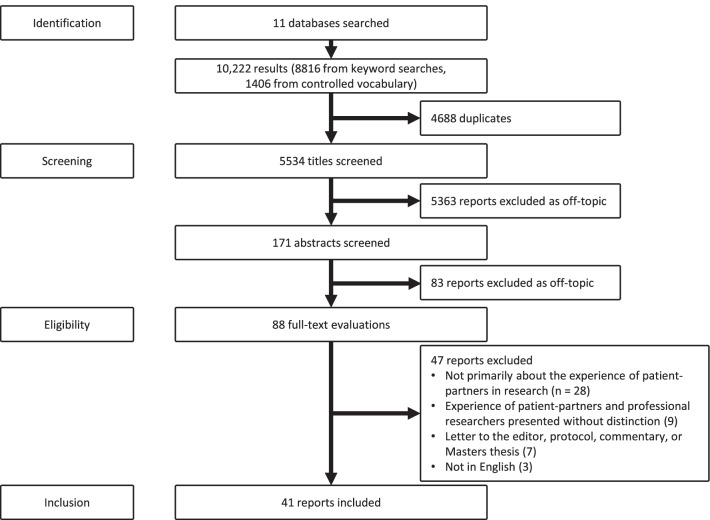
Flowchart of study selection and screening process

The electronic search strategy yielded 10,222 citations. These were imported in the *ReadCube Papers* reference management software. After elimination of duplicates, 5534 articles were screened for title and abstract and 88 full-text articles were evaluated. 41 studies were included in this review (Fig. 2).

### Study characteristics and quality assessment

Selected articles were published between 2005 and 2020, with more articles published in later years. More than half of the studies were conducted in the United Kingdom (n = 22). Others were from Canada (n = 7), the United States (n = 4), Sweden (n = 3), the Netherlands (n = 1), Norway (n = 1), Australia (n = 1), South Korea (n = 1) and Germany (n = 1). The selected studies could be divided into two groups according to their design: 21 were case studies, in which patient-partners reported on their experience within one research project, and the 20 others were cross-sectional descriptive studies that used questionnaires, interviews or focus groups to obtain the views of patient-partners with varying experiences. 7 studies were mixed-methods, and the other 34 were qualitative. Detailed study characteristics can be found in Table 1.

**Table 1:** Study characteristics [19–59]

Author, Date, Country	Aims relevant to this review (from text)	Design and sample	Demographics	Specific population	Main PPI activities
Ashcroft, 2016, United Kingdom [37]	To explore how patients and carers in eight diagnostic research specialties have been involved in research, their motivations, and the impact involvement had on them	Mixed methods with convenience sample and online semi-structured questionnaire	143 PP 77 F, 53 M Age 55-64	–	Varied between participants
Awenat, 2018, United Kingdom [38]	To investigate the ex-offender service user consultants’ experiences of being involved in the research	Qualitative case study	4 PP 2 F, 2 M Age 40-60	Ex-offenders with experience of suicidal thoughts in prison	Monthly research meetings to guide the research team
Bayliss, 2016, United Kingdom [44]	To inform the evidence base on effective ways of involving patients in a qualitative meta-synthesis.	Qualitative case study	6 PP 6 F, 0 M	Patients of rheumatoid arthritis from across Europe	Realisation of a meta-synthesis of qualitative studies
Beighton, 2019, United Kingdom [22]	To explore the perspectives and experiences of adults with intellectual disabilities and parent carers of their public and participant involvement in a health research study	Qualitative case study	9 PP (4 carers, 5 adults with intellectual disabilities) Carers: 4 F, 0 M Adults with intellectual disabilities: 2 F, 3 H Adults with intellectual disabilities: age 27-40	Adults with intellectual disabilities and parent carers	6 meetings per year: inform the choice of process and outcome measures, develop ideas for further explanatory analysis, interpret the findings of the study, disseminate results
Bench, 2020, United Kingdom [41]	To explore former patients’ and family members’ views and experiences of involvement in critical care research and/or quality improvement	Qualitative with purposive recruitment	10 PP 4 F, 6 M Age 39-78	People with lived experience of critical illness and admission to the ICU (patients and family)	Varied between participants
Bhati, 2020, Canada [48]	To report on the findings of the PERC evaluation (of patients’ experience of engagement in research)	Mixed methods with patient-partners from 3 studies in the INSPIRE-PHC	5 PP Age 60-80	–	Varied between participants
Bindels, 2014, Netherlands [39]	To investigate the required conditions, success factors, and pitfalls in potential collaborations between professional researchers and older people acting as co-researchers	Qualitative case study	3 PP 0 F, 3 M Age 60-65	Older people	Co-execution of the research project: preparing interview questions, conducting interviews, analysing the data.
Black, 2018, Canada [55]	To explore the perspectives of patients, family members, and informal caregivers who have been involved on health care research teams in Canada and elicit their recommendations for meaningful engagement	Qualitative with convenience sample	19 PP 10 F, 9 M Age 19-85	–	Varied between participants
Carlsson, 2020, Sweden [28]	To explore how persons with lived experience of a prenatal diagnosis perceived collaborating in a research project	Qualitative case study	9 PP 5 F, 4 M Age 23-43	People with experience of a prenatal diagnosis of congenital heart defect in the fetus	Regular consultative meetings
Charron, 2018, Canada [27]	To assess the community researchers’ experiences with spirometry training and their overall experiences participating in a [community-based participatory action research] project	Mixed methods case study	2 PP	Tobacco and poly-substance users, who were homeless or at-risk for homelessness	Co-execution of the research project: conception of the research question, designing questionnaires, and knowledge creation, translation, and mobilization
Coupland, 2005, Australia [29]	To explore the benefits and challenges associated with peer workers and health workers collaborating in research	Qualitative case study	4 PP Age 16-25	Young injecting drug users	Data collection: fieldwork, interviews, facilitation of focus groups. Input on the data analysis.
Damon, 2017, Canada [31]	To add to the research literature on [community-based participatory research] by drawing on the expertise of community-based ‘peer researchers’ with CBPR experience who live in the Downtown Eastside.	Qualitative with peer researchers involved in studies identified by VANDU's board of directors	14 PP 7 F, 6 M, 1 transgender Age 37-58	People who use(d) drugs	Varied between participants: participatory research.
Di Lorito, 2020, United Kingdom [25]	To propose a model for good practice in co-researching with carers of people with dementia, by reporting and synthesizing the personal reflections of the academic and lay researchers	Qualitative, case study, personal diaries, team discussion of the data	2 PP 1 F, 1 M	Carers of people with dementia	Co-execution of the research project: designing the study protocol, developing the topic guide, collecting and analysing data, and disseminating research findings
Froggatt, 2015, United Kingdom [43]	To describe the experience of PPI participation in palliative care research following a cancer diagnosis.	Qualitative with research partner representatives who had been involved in CECo research activities	8 PP 7 F, 1 M Age 57-84	Individuals with experience of a cancer diagnosis	Varied between participants
Garfield, 2015, United Kingdom [42]	To describe our experiences of lay involvement in conducting research, from both the lay observers’ and researchers’ perspectives, to inform the future role of lay people in carrying out health services research	Qualitative case study	3 PP Age over 55	–	Data collection: fieldwork
Giebel, 2019, United Kingdom [50]	To assess the extent of public involvement and explore the experiences of public advisers in the dissemination of the HHS	Qualitative case study	5 PP 3 F, 2 M	–	Writing groups (analysis and dissemination of results) and a public dissemination event
Hamilton, 2018, Canada [26]	To develop a conceptual framework for meaningful PEIR from a patient perspective	Qualitative with purposive sampling	18 PP 17 F, 1 M Age 26-68	Patients with arthritis	Varied between participants
Harding, 2010, United Kingdom [54]	To explore service users’ experiences of their involvement in developing NICE guidelines.	Qualitative with user representatives from completed or ongoing mental health GDGs	10 PP 5 F, 5M	Mental health services users	Developing NICE guidelines in mental health guideline development groups
Harrison, 2015, United Kingdom [34]	To explore the experiences of patients and carers involved in patient and public involvement (PPI) activities for stroke research.	Qualitative with purposive sampling	11 PP 6 F, 5 M Age 59-85	Stroke survivors and their carers	Varied between participants
Hemphil, 2019, United States [58]	To understand what motivates patients and caregivers to engage as partners on PCORI-funded research projects and how such engagement changed their lives	Qualitative with patient-partners of PCORI-funded research projects	255 PP 174 F, 62 M, 1 other gender, 18 missing genders Mean age 55 (SD of 14)	–	Varied between participants
Hovén, 2020, Sweden [57]	To explore the experiences of patient research partners (PRPs) and researchers engaged in a co-creative long-term collaboration in cancer research.	Qualitative case study	11 PP 7 F, 4 M Age 20-41	Cancer patients and their significant others	Regular consultative half-day meetings
Howe, 2010, United Kingdom [49]	To evaluate the efforts to ‘put principles into practice’ in public involvement research over 5 years in one specific project (PPIRes)	Mixed methods with volunteers of the PPIRes project	24 questionnaire respondents 10 PP in focus groups Focus groups: 9 F, 1 M	–	Varied between participants
Hutchinson, 2013, United Kingdom [40]	To discuss the process of working alongside people who use statutory mental health services as co-researchers	Qualitative case study	6 PP 5 F, 1 M Number of PP in age groups: 1 30-40; 2 40-50; 1 50-60; 2 over 65.	People with severe or enduring mental health problems	Co-execution of the research project: interviews, data analysis, dissemination.
Kim, 2005, South Korea [32]	To describe the experiences of researchers with a psychiatric disability	Qualitative case study	4 PP	People with a psychiatric disability	Participation in survey design, collection of survey data, presentation of survey results
Leese, 2018, Canada [20]	To examine benefits and risks in patient-partner–researcher relationships, based on patient partners’ experiences	Qualitative with convenience sample	22 PP 21 F, 1 M Age 26-68	People with inflammatory arthritis or osteo-arthritis	Varied between participants
Mann, 2018, United Kingdom [52]	To contribute evidence towards understanding how and in what circumstances PPI makes a difference	Qualitative case study	7 PP	People with multiple conditions (multimorbidity) and their carers	Regular meetings to advise on patient information leaflets, questionnaire design, ethical issues, recruitment approaches, dissemination of results
Marks, 2018, United Kingdom [51]	To share one first-time co-researcher’s reflections on the impact of PPI within a mixed methods (non-clinical trial) renal research study.	Qualitative case study	1 PP (F)	–	Commenting on and contributing to documents (protocol, interview guides, abstracts, presentations), analysis of interview data.
Matheson, 2021, United Kingdom [53]	To explore whether and how patient participation in research may promote recovery from CPTSD.	Qualitative case study	6 PP	Patients with complex post-traumatic stress disorder	Design, data collection (conducting interviews) and analysis.
McGregor, 2011, United Kingdom [56]	To explore the experience of what it is like to be a user representative in the real world.	Qualitative with "heart patients who were current user representatives"	12 PP	Heart patients	Varied between participants
Musson, 2019, United Kingdom [33]	To understand the conduct and impact that PPI can have on MND research as well as barriers and enablers to PPI by exploring the experiences of members and those who work with the SMNDRAG.	Qualitative with purposive sampling	10 PP Age 35-82	People with experience of motor neuron disease (patients, carers, relatives)	Participation in the Sheffield Motor Neurone Disorders Research Advisory Group.
Palmer, 2009, United Kingdom [23]	To discuss the process [of under-taking service user research] and to reflect on aspects of the project’s design and delivery.	Qualitative case study	5 PP 5 F, 0 M Number of PP in age groups: 3 26-35, 1 35-50, 1 51-64	Mental health services users	Co-execution of the research project: design, data collection (conducting interviews), analysis.
Reynolds, 2020, United Kingdom [19]	To answer the questions: How do PPI contributors situate their experiences of public involvement in the context of their broader lives, over time, and how are meaning and identities constructed through narratives of these experiences	Qualitative with a blend of purposive and convenience sampling	5 PP 3 F, 2 M Number of PP in age groups: 1 40-49, 2 50-59, 2 60-69	–	Varied between participants: contributed to three or more health research studies in a PPI capacity in the past 10 years
Saunders, 2016, United States [59]	To explore the experiences of AYAs and parent panel members regarding their roles as patient stakeholders in the core study	Mixed methods case study	9 PP (6 adolescents and young adults (AYA), 3 parents) AYA: 4 F, 2 M, age 17-25 Parents: 3 F, 0 M, age 50-55	Adolescents and young adults, and parents	Regular meetings in a stakeholder advisory group
Schilling, 2019, Germany [47]	To elucidate the experiences of patients and researchers who were members of a patient board that was established for a clinical trial on urinary tract infections (UTI)	Qualitative case study	7 PP 7 F, 0 M Number of PP in age groups: 2 20-34, 2 35-49, 3 50-64	Women with experience of UTIs	Participation in regular consultative patient-board meetings to provide input on the trial.
Sieck, 2017, United States [36]	To identify what both groups [researchers and patients] value about these approaches and how best to facilitate such partnerships	Qualitative with distribution of a survey to all PP of the PFEAP	72 PP	–	Varied between participants
Stuhlfaulth, 2019, Norway [30]	To investigate experiences and collaboration between patient representatives and researchers in user involvement in health research.	Qualitative with PP of the two Norwegian patient organizations	14 PP	–	Varied between participants
Thompson, 2014, United Kingdom [35]	To report on what motivated participants to get involved and their experiences of involvement in this setting	Qualitative with purposive sampling	14 PP 10 F, 4 M Number of PP in age groups: 13 55-65, 1 over 65	Cancer patients and carers	Varied between participants, each participant acting as an advisor on at least one research advisory group
Tsang, 2020, United Kingdom [24]	To describe a novel method of organizing youth participation in research and to understand the benefits and barriers of this new model	Mixed methods case study	16 PP 11 F, 5 M Age 13-25, average 19	Youth	Co-executing the research: the creation of project protocols, questionnaire design, ethics applications, and project execution through the organization of focus groups
Vanderlee, 2020, Canada [46]	To examine the experiences of researchers and parents of children with a neurodevelopmental condition who participated on a research study advisory committee	Qualitative with purposive sampling	6 PP	Parents of children with a neurodevelopmental disability	Participation in a parent advisory committee
Warner, 2021, Sweden [21]	To describe the immediate impact of PPI from the user representatives’ perspective in a case study of refugee involvement in the development of mental health intervention research.	Mixed methods case study	4 PP	Refugees with experience of children experiencing post-traumatic stress	One‐day meeting for group discussion on the trial design
Young, 2019, United States [45]	To examine the relationships that developed between investigators and patient-partners over 18 months.	Qualitative with PP of the VPPRN	13 PP	PP related to the Vasculitis PPRN	Participation in the VPPRN governance.

*PP* patient-partner, *F* female, *M* male

Critical appraisal suggested that most studies were of adequate quality. However, 14 studies failed to take the relationship between the researchers and study participants into account (CASP qualitative checklist criterion 6), and 10 studies omitted to describe the data analysis methods (criterion 8). The other criteria of the CASP qualitative checklist were fulfilled by most studies. Five studies [23, 24, 27, 36, 40] were judged to be of lower quality, i.e., they were evaluated to fulfill less than 8 criteria (≤ 7/10). The unfulfilled criteria were mostly not reported on, rather than judged to be unsatisfactory. Of the five studies, four were case reports on participative methods [23, 24, 27, 40]. In these articles, the methods of data collection and analysis of the patient-partners’ experiences were unreported, as the “Methods” section focussed on the participative methods of the project described. Similarly, the fifth study [36] did not report on the methods of analysis. The complete results of the quality assessment can be found in the Additional File 3.

### Thematic analysis

**Fig. 3:**
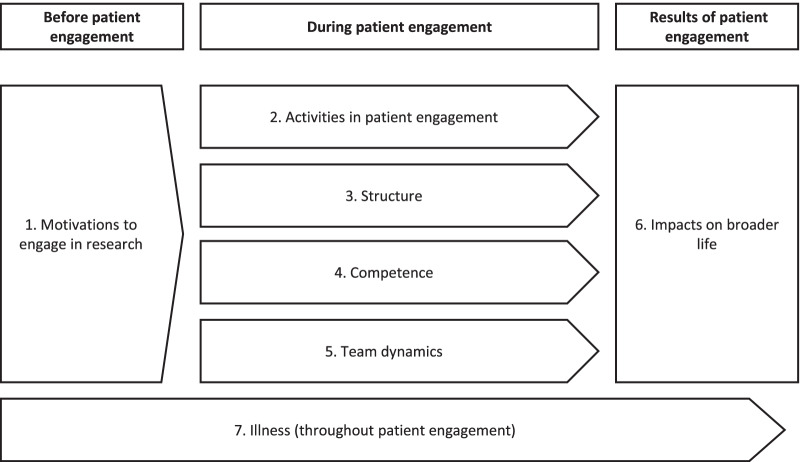
Visual organisation of the themes

The thematic analysis identified 7 main themes. Figure 3 presents a visual organisation of these themes and has been constructed to place them in a logical order and to provide a global understanding of the results. *Motivations to engage in research* describes *why* patient-partners get involved in the first place. *Activities in patient engagement* describes how patient-partners experience *what* they do in research. *Structure* describes how patient-partners experience *how* patient engagement is implemented. *Competence* describes how patient-partners perceive their ability to engage in research and how this affects their experience. *Team dynamics* describes how patient-partners experience *with whom* they work. *Impacts on broader life* describes how patient engagement affects other spheres of patient-partners’ lives, often as a result of their experiences in the themes previously named. Finally, *Illness* describes how health issues taint patient-partners’ experiences throughout their engagement. In the following sections, quotations were chosen to broadly represent the themes in which they were found. Quotations that are the direct words of a patient-partner are identified by a note in brackets.

#### Motivations to engage in research

The most frequently reported motivations to engage in research were divided into three subthemes: altruistic motivations, personal motivations, and motivations related to illness. Social motivations were less frequently reported but were important to some patient-partners [40, 49, 56–58].

“I am retired and 77, and because I don’t have family or many friends it is nice to be able to sit on committees. […] It is social integration for the elderly!” (PP) [56]

##### Altruistic motivations

The most commonly described motivations were related to a desire to help [19, 24, 26–33, 35, 37–39, 41, 48, 49, 55–58]. Specifically, patient-partners wanted to contribute to better research [26, 32, 33, 48, 49] and to improve healthcare [19, 26, 28, 32, 33, 35, 39, 48, 49, 55–58], most often with a general desire to help other patients [19, 26–29, 31, 33, 35, 37–39, 41, 49, 55–58] or their communities [27, 29–31, 58]. The benefit of others was often described as the ultimate goal of patient-partners, while improving care and research were seen as the means to this end [26, 35, 38, 39, 49, 55, 58].

“I want to contribute to this research project because I want to help improve the quality of life of others in need and I believe research is the best mechanism to do so” (PP) [58]

Furthermore, many patient-partners described engagement in research as a way to have their voices heard [19, 21, 26, 32, 33, 35, 37, 49, 57, 58]. This activistic motivation was often described as a desire to represent the broader patient population [19, 32, 35, 37, 49] and was sometimes legitimized by the idea that the public has a right to influence research [19, 33, 49, 58].

“Many people set out to ensure patients and/or carer perspectives were explicitly represented, to shape research into what patients and carers want.” [37]

##### Personal motivations

Personal benefits were less frequently described as a reason for engagement than altruism but remained a prevalent theme [19, 26, 29, 30, 32–35, 39, 49, 55, 57, 58]. Codes under this subtheme often described the idea that patient-partners engaged in research because it was “a fun activity” [57]. For example, an interest in research [19, 33, 39, 49, 58] and the desire to participate in a stimulating activity [19, 26, 34, 49, 57] were commonly cited motivations.

“She also positioned herself in opposition to this [altruistic motivations], often casting her involvement in PPI as “fairly selfish” because it satisfies her personal “curiosity” and helps to keep “the mind going.” [19]

Engagement in research was also seen as an opportunity for personal or professional development [19, 29, 30, 32, 33, 35, 49, 57, 58]. Indeed, some patient-partners hoped to learn skills and acquire knowledge through their engagement. For example, one study about the experience of injecting drug users engaged in research mentioned:

“When the PWs [peer workers] were approached to participate in the research, they were all keen to be involved to gain work experience.” [29]

##### Motivations related to illness

Multiple studies highlighted illness as the starting point to engagement in research for many patient-partners [19, 26, 33–35, 37–41, 47–49, 55–59]. A central idea in these studies was that patient-partners wanted to work on something relating to their health experiences [19, 26, 34, 35, 38, 39, 47–49, 56, 58]. Within this theme, two distinct situations were common: some patients were grateful for the adequate care they had received and wanted to “give back” to the medical community [19, 33–35, 40, 41, 56, 59]; others were frustrated by the negative experiences they had had and wished to improve the system for future patients [19, 35, 39, 41, 49, 55–58].

“I was so impressed with [cardiac] rehab that I wanted to give something back.” (PP) [56]

“The reason I got into the volunteering is to make the experience better than it was for me in those early days so that...the new families that are coming up won’t have such a broken experience.” (PP) [55]

Another motivation was the “desire to turn something negative into something positive” [41]. Through engagement in an activity which turned their illness into an asset, patient-partners hoped to “gain personal meaning from the experience of illness” [37]

#### Activities in patient engagement

Many articles described patient-partners’ appreciation of the tasks they undertook in research. It was often said that activities were seen as “generally positive” [26, 36], while generally negative experiences were rarely reported.

##### Intellectual stimulation

Engagement in research was characterized as an interesting and stimulating activity [19, 25, 34, 35, 37, 42, 43, 52, 55, 57]. Two factors were described as contributing to these qualities: the challenges encountered [23, 34, 35, 51, 52] and the opportunity to learn [19, 21–27, 29, 31–35, 37–39, 41–44, 46–53, 55, 57–59].

Challenges in tasks arose from the novelty of research methodology [28, 51], the complexity of topics [23, 28, 34, 59], and the intensity of activities [19, 33, 45, 47]. This last challenge was seen in long meetings, for which sustained attention was necessary [33, 47]. For some patient-partners these challenges were positive, as they allowed them to remain engaged and to push their limits [23, 34, 35, 51, 52].

Alan spoke of his “enjoyment and intellectual stimulation from the challenges of research” [35]

For others, however, challenges were overwhelming or exhausting, and more support would have improved their experiences [23, 28, 32, 34, 59].

“I tend to speak a bit less because the really technical subjects are more tricky so I’m listening very hard to think what they’re actually talking about … sometimes at those meetings I feel rather out of my depth.” (PP) [34]

The opportunity to gain knowledge and skills was universally appreciated across studies [21, 23–27, 29, 31, 33–35, 37, 41, 43, 44, 47, 50–53, 55, 57–59]. Many patient-partners reported that engagement in research had allowed them to learn about research, healthcare, and their illness [19, 24, 33, 35, 47, 51, 52, 57, 59].

“Being a research partner was a chance to gain information about their cancer and a wider perspective on cancer research in general.” (PP) [43]

Furthermore, a range of abilities were developed in patient engagement, such as skills in research, communication, and leadership [24, 26, 27, 29, 31, 33, 35, 37, 38, 43, 44, 50, 53, 55, 58]. Overall, learning was seen as valuable for its own sake and as a useful way to improve patient-partners’ self-care and understanding of others.

“It gave me further insight into others’ lived experiences, enabling me to prepare for a future, which could see me on a parallel journey.” (PP) [25]

##### Impact of patient-partners

A desirable aspect of patient-partners’ experience was “feeling useful” [37]. Concretely, this was associated with their perceived impact on research projects. In most articles, patient-partners felt that their contributions had had an impact [19–22, 24, 25, 28, 30–34, 37, 38, 41, 43, 44, 46–48, 51, 52, 54, 57, 59], but in others, they felt that they did not significantly influence research [25, 30, 31, 37, 39, 44, 52, 54, 56]. The latter situation was often linked to dismissive or closed-minded behaviors of professional researchers [25, 31, 52, 54, 56], which are discussed at greater length in *team dynamics*.

“It is like 10 reasons why things can’t happen and no reasons why things can. Because, we basically go [to the meetings] and listen.” (PP) [56]

Factors enabling meaningful contributions related again to researchers’ attitudes [20, 22, 38, 41, 57], but also to the pertinence of activities [23, 25, 31, 36, 37, 39, 41, 44, 45, 47, 56, 57, 59]. Activities for which patient-partners were well prepared [41, 44, 45], which were clearly structured [41, 57, 59], and which focussed on practical issues [39, 44, 47, 59] were said to lead to more significant engagement. When patient-partners felt that they participated meaningfully, they reported a sense of usefulness and of contribution to society and to other patients [23, 25, 26, 28, 37, 39, 41, 46, 48, 51, 57–59].

“It is important that I am able to contribute to society in general, and it gives me a feeling of joy and satisfaction.” (PP) [39]

“It also feels great to have a hand, however small, in making a change or a difference to people’s lives, being stigmatised is nasty, lets help it stop.” (PP) [23]

It was rather commonly reported that patient-partners were simply unable to perceive their influence on research [33, 41, 43, 44, 47, 49, 50, 55]. As patient-partners appreciated when professional researchers validated their impact [20, 25, 28, 33, 34, 38, 39, 55, 57], cases where no feedback was received were strongly criticized.

“Follow up after a study is completed is something that is sorely lacking… I think it is a massive way in creating value for people participating in the research… Just show them that their efforts weren’t in vain…” (PP) [55]

On the other hand, when the contributions of patient-partners were adequately recognized, it made them feel valued, respected, and important [20, 25, 28, 33, 34, 38, 39, 55, 57].

“Participants valued researchers who provided reassurances that they deemed patients’ contributions to be important. For many participants, these reassurances strengthened their sense of feeling genuinely respected in their relationships with researchers, which helped them feel at ease to contribute.” [20]

#### Structure

How patient engagement was structured significantly influenced the experience of research. This theme highlighted the importance for patient-partners to understand the context of their involvement, to choose their level of engagement, and to participate in a way that was convenient for them.

##### Making things clear

Articles widely stated the importance of clarity in patient engagement [19–22, 25, 26, 30, 32, 33, 36, 37, 41, 44–47, 49–51, 54–57, 59]. Particularly, patient-partners felt that they should receive enough information concerning their roles in the project [26, 30, 33, 37, 41, 44, 45, 47, 50, 51, 55, 56, 59]. This was often reported to be a shortcoming of patient engagement: many patient-partners were unsure of what was expected of them [26, 30, 33, 37, 44, 45, 47, 50, 51, 55, 56, 59].

“Some public advisers felt unsure after some meetings why they had attended the meetings.” [50]

In addition to clear roles, patient-partners wanted to have a global view of the project [20, 36, 41, 45, 49, 50, 59].

“Almost a kind of, underground map of the different stages explaining…where we’re at, where you’ll be involved, where you get on, where you get off and clearly to just kind of help just help visualize the view from the patient’s involvement.” (PP) [41]

When researchers managed to make these features clear, patient-partners found it easier to navigate the project and to contribute meaningfully [25, 41, 45, 47, 49, 55, 57].

##### Level of engagement

When discussing this theme, articles often stressed that there is no rigid recipe for engaging patient-partners: all patient engagement should be individualized to each project and each person [19, 20, 26, 28, 33, 34, 36, 41, 46, 51, 55, 57].

This was illustrated by the large variation in the satisfaction of patient-partners regarding their degree of engagement: some believed they were insufficiently involved [19, 28, 30, 31, 34, 37, 39, 45, 47, 49, 52, 56, 57], while others were satisfied with their participation [20, 22, 26–28, 31, 33, 34, 36–38, 41, 46, 48, 51, 52, 56, 57], and yet others were overwhelmed with their engagement [20, 34, 37, 46, 51]. These levels of satisfaction did not generally correlate with the level of engagement, e.g., patient-partners acting in co-production [4] (more engagement) were not more satisfied than those acting in a collaborative model (less engagement). [4] However, many patient-partners mentioned that high levels of engagement were something to strive for [19, 22, 28, 34, 37, 39, 41, 45, 47–49, 51, 56, 57].

“They were emphatic that they would like to be involved from inception of any research study, taking more of a ‘control’ approach.” [22]

Many patient-partners noted that it was necessary to accommodate people with different availabilities and different motivation levels [20, 26, 33, 41, 46, 55, 57], and that it was important to consider the views of those who had fewer opportunities to engage [28, 34, 55]. They rationalized this suggestion by stating that this enabled a broader range of people to engage in research [28, 55].

“At one focus group meeting, a participant commented that the research environment was impacted by the participation of ‘super patients’, who have more time to dedicate to volunteering on health care teams, and whose voices may get counted more often.” [55]

Flexibility was also valuable for patient-partners: they appreciated projects in which the workload could be varied according to fluctuating constraints in their lives [20, 33, 36, 46].

“Participants valued relationships with researchers who recognized they were managing their role as patient partners among multiple obligations, priorities and their own health.” [20]

##### Logistics

Many logistical issues were discussed in the data: time and energy constraints, health issues, modality or location of meetings, transportation and parking, and financial constraints, among others. When discussing logistics, articles agreed on one point: convenience is a prerequisite to engage patients [20, 26, 28, 30, 33–36, 41, 42, 47, 50–52, 55, 57, 59]. When logistics remained a challenge for patient-partners, they either could not participate, or it led to stressful experiences [23, 34, 41, 42].

“The more constraints you put in… then they drop out.” (PP) [41]

Regarding payment, some patient-partners expected their expenses to be covered [19, 26, 33, 49, 55, 57], some expected to receive a salary [19, 26, 34, 37, 49, 55, 57], and some did not expect any payment [33, 37, 41, 47]. When they did receive compensation for their work, patient-partners were appreciative as they felt valued [19, 26, 33, 47, 49, 55, 57, 58]. Monetary compensations were also seen as a way to include people for whom financial issues were a barrier [19, 37, 41, 55].

“In the second research team I’m involved with I get an hourly wage for the work that I do. So, I feel, you know, I am a fully valued member of this team...” (PP) [55]

“It’s not that I want to personally make money doing this… It’s respect.” (PP) [26]

#### Competence

Almost all studies described patient-partners’ reflections about their perceived abilities to participate in research projects. Patient-partners’ competence was an important topic because it lay the foundation to power differentials in research teams, which are discussed in *team dynamics*. Two types of competence were described for patient-partners: experiential competence and competence from skills.

Few articles discussed patient-partners’ views on the abilities of professional researchers to use patient engagement. When it was discussed, experiences varied, with some patient-partners reporting that professionals were well prepared [45, 56–58], but others stating the contrary [34, 37, 45, 55].

“Preparation was beneficial and recommended, both for PRPs [patient research partners] and for researchers, who often seemed unprepared for working with PRPs.” [55]

##### Experiential competence

Experiential competence was described as applicable knowledge drawn from experiences with health issues or other related experiences [19, 21, 25, 28–31, 33, 34, 36, 41, 43, 46, 51, 52, 54, 55, 57–59]. As this is what patient engagement seeks to add to research, most patient-partners were confident that their experiential competence was useful and complemented the qualifications of professional researchers [19, 21, 22, 25–36, 38, 41, 43, 46, 48, 51, 52, 54, 55, 57–59].

“The most frequently described beneficial impact of PPI to the research process was bringing a different perspective. Participants thought the synergy of the experiential expertise of stroke survivors with the professional expertise of researchers and clinicians benefitted the research process.” [34]

However, there were instances where patient-partners were unsure that their experiences were useful, which led to the feeling that they did not belong in research [33, 53, 54, 56]. While this situation was rare, it occurred most often when patient-partners compared themselves to professional researchers [54].

“I thought ‘here are all these very professional people, the experts . . . and there’s little old me that’s had this experience and it was a long time ago.” (PP) [54]

##### Competence from skills

Studies reported that patient-partners used many other skills in patient engagement, such as writing and language abilities when adapting documents for participants, or social skills when leading interviews for data collection [25, 26, 35, 37, 39, 42, 46, 51, 53–55]. Some studies even proposed that certain abilities were necessary for patient-engagement, such as communication skills, critical thinking, and confidence to contribute [25, 26, 30, 33, 34, 40, 54, 56, 59]. Research-related skills were also discussed. Patient-partners generally agreed that they needed a basic understanding of the research process to be able to contribute [23, 28, 33, 44, 51].

“A common belief among members and researchers was that there is a “certain type of person” needed for PPI. However, no one explicitly said what this kind of person was. Inferred properties appeared to be someone who would be well-educated and confident with research and participating in groups.” [33]

Contrary to experiential competence, it was common for patient-partners to lack confidence in their skills and their knowledge of research [20, 23, 25, 26, 28, 29, 34, 36, 44, 50, 55, 56], which often led them to refrain from contributing, out of fear that they might “sound stupid” [55]. The lack of qualifications was a major factor that contributed to the feeling of inadequacy, especially when compared to the qualifications of professional researchers [20, 23, 25, 29, 44, 54, 59].

“Many participants had perceived themselves as lacking experience, knowledge or credentials, which they expressed prompted them to feel inadequate or insecure when they were interacting with (or contemplating the possibility of interacting with) researchers.” [20]

##### Training

In most studies discussing training, patient-partners reported that it was appreciated and desirable [23, 25, 26, 30, 32, 34, 36, 38, 40, 41, 43, 44, 48–51, 55]. Patient-partners who felt that they had received enough training reported that it enabled them to make more practical contributions, understand the projects in which they participated, and be more confident in their abilities [23, 25, 26, 33, 34, 40, 44, 49, 51, 55].

“The training sessions were enormously helpful in reiterating the fact that I still had a brain!” [40]

However, some patient-partners felt that they had not received enough training, which undermined their confidence [23, 30, 32, 44].

“I would have liked to go together with the researcher (face to face) through one page or so from the materials to be coded so that I am confident I’m doing the right thing.” (PP) [44]

A small but significant proportion of studies reported that patient-partners did not want to receive training [33, 34, 41, 48, 59]. These patient-partners were usually very confident in the value of their experiential knowledge, and some stated that training could professionalize them, which would “detract from their lay role”. [34]

“Everybody there had experience of a stroke or being a carer for somebody, so in a way, that was the training you could say, yes.” (PP) [34]

#### Team dynamics

The analysis suggested that team dynamics had the potential to make or break the experience of patient-partners. The most influential factor in this theme was the power differentials that could be perceived between professional researchers and patient-partners. Many facilitators and barriers to a positive team atmosphere were identified, and it was often noted that team dynamics change as relationships are built.

### Power balance

Most articles recognized the potential for power differentials to exist between professional researchers and patient-partners [19–21, 25–31, 33, 34, 37–39, 42, 46, 51, 54, 55, 57, 59]. Even though potential power differentials were often discussed, it was most often reported that patient-partners felt that they were on equal grounds with other team members [20–22, 25–28, 33, 34, 36, 38, 39, 46, 47, 49, 51, 52, 57, 59]. Nonetheless, patient-partners sometimes felt that there existed a hierarchy in research teams, where they were less influential than professional researchers [19, 20, 27, 29–31, 34, 54, 55, 59]. These power differentials were found to be based on perceived or real competence differentials [19, 27, 30, 55, 59].

“Another participant commented on inequity within research teams: “I think there is this real sense of a power imbalance when you’re sitting around a room with...10 or 15 other individuals, all of whom have the title ‘doctor’...”” (PP) [55]

When discussing this issue, patient-partners stated that considerable effort should be invested to reduce the power differentials, as this greatly impacted their experience [20, 26, 33, 37, 39, 41, 51, 54]. When power balance was achieved, patient-partners often attributed this to enabling attitudes of professional researchers who ensured that all members of the team felt heard and respected [20–22, 25–28, 33, 34, 36, 38, 39, 46, 47, 49, 51, 52, 57, 59].

“A positive and welcoming research environment fosters a feeling of connection with no emphasis on maintaining power or hierarchical differences among members.” [26]

Projects in which hierarchies were abolished led patient-partners to feel empowered to contribute [20, 21, 26, 31, 33, 39], to feel respected [20–22, 27, 28, 31, 33, 34, 36–39, 46, 47, 51, 52, 54, 59], and to feel “part of the team” [20, 21, 23, 25, 28, 33, 39, 42, 46, 51, 55, 57, 58]. However, instances where hierarchies remained were highly criticized and led patient-partners to “[express] frustration and resentment, [feel] ‘disheartened’ and [seek] to ‘wash their hands’ of the project” [31].

### Barriers and facilitators

When patient-partners discussed barriers and facilitators to positive team dynamics, they often focussed on the attitudes of professional researchers. For example, identified facilitating behaviors were openness to patient-partners’ contributions [33, 36, 38, 41, 45, 47, 49, 52, 55, 57], inclusivity of all patient-partners [21, 26, 33, 34, 41, 44, 52, 54, 57], open communication [20, 26, 36, 38, 39, 41, 54], and supportive attitudes [19, 20, 23, 26, 29, 32–34, 37–39, 41–43, 46, 47, 50, 55, 56].

“They’ve always listened, they always take their time – they don’t butt in; they always let you finish… They don’t try to put words into your mouth. It’s what you say is what they hear.” (PP) [38]

A significant number of studies identified situations where professional researchers’ attitudes had acted as barriers [19, 20, 29, 31, 37, 38, 41, 55]. Notably, dismissive approaches [19, 25, 29, 31, 37, 41, 54, 55], judgemental attitudes [19, 38, 41, 55], unprofessional behaviors [19, 41, 56], and unwillingness to connect with patient-partners [25, 31, 34, 41] were described.

“There was no give and take. It was just, ‘Do this.’ You know, ‘Take these questionnaires and fill ‘em out, then hand them back in.’ That was it. There was no input from us at all.” (PP) [31]

Few barriers or facilitators were identified on the patient-partners’ part. The most frequently identified barrier was that some patient-partners had negative expectations of professional researchers that arose from negative past experiences or beliefs [38, 46].

“Many parents [patient-partners] had assumed that research was “academic” and “all business.”” [46]

### Relationships

Many relationships were built during patient engagement [19, 20, 24, 26, 28, 33, 34, 37–39, 42, 45, 46, 49, 51, 52, 55, 58] and patient-partners often wanted to know their fellow team members beyond their patient engagement roles, on a personal basis [19, 20, 23, 30, 39, 46, 55].

“I think one of the nice pieces was that the researchers were flexible with sharing on Facebook pages and just getting to know us as families. And see our pictures of families on holidays and at school and that type of thing.” (PP) [46]

Building relationships, though desirable, was sometimes seen as a challenge [19, 20, 35, 45], especially in the early stages of getting introduced to the group [20, 26, 30, 33, 41]: “There’s a fear when you start…” [26] Relationships were often developed in informal social activities or during meals when patient-partners could simply enjoy conversations with each other and with professional researchers [19, 20, 23, 26, 33, 55].

“[W]e have summer lunches and Christmas dinners, it’s informal Christmas get together and just to talk about where’re [*sic*] you going, what have you done, are you done with Christmas shopping things like what you would talk about with a close friend, kind of conversation. Yeah, lots of joking around, it’s fun.” (PP) [26]

In addition to the intrinsic value of relationships, studies suggested that close connections between team members enabled meaningful patient engagement by enhancing trust, allowing patient-partners to feel comfortable sharing their experiences, and contributing to bridge power differentials [20, 21, 28, 30, 41, 46, 47, 59].

“A need for trust between collaborators was seen as necessary before feeling comfortable to disclose personal perspectives.” [28]

#### Impacts on broader life

Most patient-partners indicated that engagement in research was valuable to them because it had positive effects on their lives [19, 21, 25–29, 31, 35, 37, 38, 40, 42, 50, 57, 58]. Psychological and social impacts were identified. For some patient-partners, research became ingrained in their identity.

### Positive psychological impacts

In the majority of cases, patient engagement was reported to have positive psychological effects. Patient-partners found it gratifying to see the results of their contributions [19, 23, 25–28, 32, 33, 37, 51–53, 55]. They were proud to have a part in the improvement of other patients’ care [19, 25, 28, 38, 41].

“I’m happy to contribute and be part of the solution that could help others who are suffering with the disease. It’s [a] very fulfilling feeling.” (PP) [26]

As discussed in the theme *Competence*, some patient-partners reported that being part of a professional academic team was a validation of their special experiential competence [21, 27, 38]. This, combined with the recognition of their contributions by professional researchers, led patient-partners to feel important and valued [21, 23, 28, 29, 33, 34, 37, 38, 40, 41, 46, 52, 56, 57].

“To be in university and to be with other lecturers… and to be treated like – we have honour passes [identity card]… it makes you feel special like you… are appreciated by the university.” (PP) [38]

In many cases, patient-partners reported that these external validations increased their self-esteem and confidence [21, 23, 25, 27–29, 31, 33, 38, 53]. This was particularly meaningful in studies on mental health:

“I didn’t want to go home. Every day I learnt something new. It got my mind off things and it made me feel like I’m someone. [Before] I thought if I die, I’m nothing, nobody’s gonna notice. Now I know the world cares.” (PP) [29]

### Negative psychological impacts

Negative psychological impacts were seldom reported. It was noted, however, that projects in which patient-partners did not feel useful or competent did not lead to the above-mentioned positive impacts. Furthermore, the instances where patient-partners were overwhelmed with research tasks led to feelings of exhaustion and stress [20, 37, 46, 51].

“[There is a] constant guilty, nagging feeling that I’m not doing enough… I have thought about [stepping down from being a patient partner] for that reason…” (PP) [20]

It was also found that patient engagement had the potential to cause distress among patient-partners, especially when the project touched on sensitive topics [22, 23, 25, 28, 33, 40, 41, 43].

“One interview brought back some uncomfortable issues... I felt very tearful and distressed afterwards. Although we talked about it I didn’t think that it would happen and when it came up I wasn’t ready for it.” (PP) [23]

### Social impacts

Patient engagement allowed many patient-partners to build social networks [19, 24, 26, 30, 33, 34, 37, 38, 40, 41, 50, 52, 55, 56, 58]. Being part of a group, meeting new people and making friends were seen as important benefits of patient engagement [19, 20, 24, 26, 28, 30, 33, 34, 37, 38, 40, 41, 46, 49, 50, 52, 55, 56, 58].

“There’s one gentleman that’s lost his wife and he’s very grateful and happy to be part of the group actually because he, for one thing, it gets him out, he’s socializing…so he’s happy to be there too, for the social side.” (PP) [41]

For many patient-partners, engagement in research was a valuable opportunity to meet people with similar experiences of health problems [27, 28, 33, 34, 37, 41, 43, 46, 48, 53, 57, 58]. Through these new connections, patient-partners had the opportunity to share about their illness and to learn from each other [33, 34, 37, 40, 41, 43, 48, 51, 52, 58].

“There was also a dimension of support that individuals gained from their involvement. Some participants referred to the value of being able to talk about their illness to other people who had experienced cancer.” [43]

### Identity

It was reported that many patient-partners started patient-engagement in a context where they had stopped their professional activities, either due to health issues or because they were retired [19, 35, 37, 55, 58]. For these patients, engagement in research provided them with the opportunity to participate in a meaningful activity [19, 35, 37, 38, 55, 58].

“I’ve had to give up work and it [patient engagement] was another way of giving meaning to life, really (…)” (PP) [35]

Other patients, especially those with experiences of mental health problems or substance use, reported that engagement in research led to new opportunities, such as professional activities or higher education [24, 29, 31, 37, 38, 58].

“PPI has given me the experience and courage to try new things, so much so, that I am actually taking an MSc in Social Work.” (PP) [37]

At higher levels of engagement, many patient-partners reported that patient engagement became a part of their identity [19, 26, 31, 35, 37, 38, 41, 58]. They were no longer simply people who engaged in research, they were *patient-partners*.

“It’s not every ex‐offender who has that same output or outlook… because I know other ex‐offenders and they’re still doing whatever it is that they do. … whereas for us, I think that with each and every one of us who is part of this group, we have all changed our lives in a positive way by being part of this group.” (PP) [38]

“This project has helped me to expand my thinking…to redefine who I am, and work towards the goal of making it my career.” (PP) [58]

#### Illness

While illness experiences varied between patient-partners, health problems often interacted with patient engagement. Bidirectional effects were found, where illness affected patient engagement, and patient engagement affected illness. It was noted that this theme was more important for patients with more pervasive illness experiences.

### Illness affects patient engagement

Many articles described barriers to engagement that emerged from illness [19, 20, 26, 33, 34, 41, 46, 52, 59]. For example, illness could present many logistical challenges, such as time constraints related to healthcare [41, 46, 52, 59] and issues with transport due to mobility handicaps [19, 33, 34, 41].

“I can no longer drive because of my stroke.” (PP) [50]

Functional barriers were also found [20, 26, 33, 34]. Notably, low energy levels were a barrier to long meetings and activities [33, 34]. In some cases, health posed such a challenge that engagement had to be reduced [20, 26, 33, 59].

“[P]eople who are involved have different medical conditions including arthritis… You know if they had a big exacerbation of their symptoms and need to pull back or pull out, that flexibility must be part of the [study] organization.” (PP) [26]

On the flip side, illness could be a motivator to engage in research, as was discussed in *motivations* [19, 26, 33, 37, 41, 49].

“After 6 months of “sitting indoors,” Brendan identified having a “light bulb moment” when attending a postdiagnosis support event, which motivated him to do as much as he could for as long as possible.” [19]

### Patient engagement affects illness

Patient engagement was said to promote health among patient-partners, as it led them to learn about their illness and enabled them to take better care of themselves [19, 35, 58, 59].

“For example, partners noted learning new skills or mastering technology to manage their health, increasing medication adherence, visiting their clinician more often, improving their use of preventive care and asking more questions or seeking more information about their care.” [58]

In some cases, patient engagement could even promote recovery, such as in mental health or in stroke patients [31, 34, 37, 40, 41, 53].

“And of course, given I had a stroke I can quite literally try and get my grey matter to work again.” (PP) [34]

While direct impacts on illness were only seen in some populations, most patient-partners agreed that engagement in research could improve their experience of illness [19, 26–28, 33–35, 37, 38, 40, 41, 43, 46, 48, 51, 53, 57–59]. For some, the opportunity to share with other patients helped in accepting or moving on from illness [19, 28, 35, 37, 38, 40, 41, 57].

“Venting their feelings and experiences together with a group of peers with similar experiences was considered an emotional relief and felt liberating.” [28]

Engagement in research led some patient-partners to find meaning for their illness [19, 28, 35, 37, 38, 57], as they developed “a sense that something positive had come out of the negativity of diagnosis and treatment” [37]. Finally, patient engagement allowed some patient-partners to gain agency on their illness [26, 33, 35, 59].

“An arthritis diagnosis can make you feel powerless but collaborating with researchers that listen to and appreciate your feedback gives you some of that power back.” (PP) [26]

#### Positive and negative experiences

The seven themes identified both positive and negative experiences, the most important of which are listed in Table 2 for a concise view of the successes and pitfalls of patient engagement. As shown in this table, some aspects of patient engagement can lead to opposite experiences depending on their success or failure. Other aspects were only reported upon through either positive or negative experiences. Blank boxes were left where no opposite experience was reported.

**Table 2:** Positive and negative experiences in patient engagement

Theme	Positive experiences	Negative experiences
1—Motivations to engage in research	Project related to patient-partners’ experiences	
2—Activities in patient engagement	Learning and developing skills	
	Stimulating challenges	Overwhelming challenges
	Feeling useful because impacts of contributions are perceived	Feeling useless because no impact is perceived
		Not knowing if contributions have an impact
3—Structure	Clear roles and project progression	Unclear roles and project progression
	Adequate engagement level	Engagement level too low
		Engagement level too high
	Flexible engagement level	
	Convenient logistics	Inconvenient logistics
4—Competence	Confidence in competence	Low confidence in competence
5—Team dynamics	Power balance achieved	Power differentials maintained
	Informal atmosphere	
	Positive researcher attitudes	Negative researcher attitudes
	Relationships beyond patient engagement roles	Few meaningful relationships
6—Impacts on broader life	Pride from engaging	
	Validation that patient-partners are important	
	Increased self-esteem	
		Distress through sensitive topics
	Building a social network	
	Peer support for illness	
	Participation in a meaningful activity, development of identity	
7—Illness	Illness-related logistical challenges considered by researchers	Illness-related logistical challenges not considered by researchers
	Better health	
	Finding meaning for illness	

Positive and negative experiences on the same line represent opposite experiences of the same aspect. The relative frequency of these opposite experiences is discussed in the corresponding theme’s section

## Discussion

The objective of this review was to synthesize the qualitative literature describing the experiences of patient-partners in research. The 41 selected articles and 7 themes identified provided a detailed portrait of this experience and highlighted positive and negative aspects of patient engagement, as perceived by patient-partners.

The quality appraisal identified 5 studies of lower quality [23, 24, 27, 36, 40], where less than 8 criteria (< = 7) of the CASP qualitative checklist were fulfilled. Despite these unfulfilled criteria, the authors of this review believe that all selected studies were relevant to the analysis. Indeed, all studies were published in peer-reviewed journals, and all studies were judged to be faithful descriptions of the experiences of patient-partners. Nevertheless, the reader should keep the results of the CASP qualitative checklist evaluation in mind when considering the findings of the thematic analysis.

Though the seven emergent themes represent distinct aspects of patient engagement, one common thread can be described to generalize the findings of this review: for patient-partners to have a positive experience, they must feel that they belong in research. Patient-partners who feel out of place will generally have worse experiences and engage less meaningfully than those who believe that their presence and contributions are important to studies. The latter, through a stronger sense of being part of the research team and the project, have more fulfilling experiences. Perhaps the factor which most directly affects whether patient-partners feel in their place is their perception of the impacts they have on research. Patient-partners who can see the results of their contributions have proof that they serve a purpose in research, and this may explain why validation of their impacts is so desirable, as described in *activities in patient engagement* [20, 25, 33, 36–38, 41, 44, 48, 49, 55, 57]. When this feeling of belonging is achieved, the positive psychological impacts described in *impacts on broader life* are enhanced: engagement in research becomes a highly gratifying, validating, and empowering experience.

Through the identification of strengths and weaknesses within patient engagement, the authors generated a list of recommendations to improve patient-partners’ experience (Table 3). Many specific recommendations were proposed in the selection of articles; however, the proposed solutions presented in Table 3 arose from what was reported as the most important factors influencing patient-partners’ experiences. Table 3 is therefore constructed to reflect this process, with the findings that led to the recommendations paired with the recommendation.

**Table 3:** Authors’ recommendations to enable positive PP experiences

Finding (summarized)	Recommendation
Some challenges can be overwhelming to PPs	Provide adequate support to PPs in their tasks
PPs who can perceive the impacts of their contributions feel useful, whereas those who do not feel useless	Provide feedback to PPs about their contributions
PPs who receive external validation of their contributions feel valued	
Clear roles increase PPs’ confidence	Ensure that PPs understand their roles
	Explicitely state the purpose of PPs’ presence to all activities
PPs who can decide their level of engagement feel respected	Discuss PP’s desired level of engagement
	Be flexible with PPs’ level of engagement over time
Convenient logistics enable PE. Inconveniences lead to PPs dropping out	When organising PE, consider PPs’ constraints related to other commitments, illness, transport, and financial issues
PPs who are confident in their competence are enabled to contribute to PE, whereas those who do not feel anxious	Provide training or preparation for research activities for PPs who desire these
Power differentials lead PPs to feel less valued and less useful	Adopt proactive behaviours to abolish power differentials
Professional researchers’ attitudes are an important factor affecting team dynamics	Be open, inclusive and respectful to PPs
Informal team atmospheres enable power balance	Organize informal social activities, such as team meals
Meaningful relationships between team members lead PPs to feel valued and enables trust	

*PE* patient engagement, *PP* patient-partner

Other reviews have touched on the experience of patient-partners in research, but to the authors knowledge, this is the first review to focus specifically on this topic. A systematic review by Brett et al*.* [60] described impacts that patient engagement had on patient-partners, many of which align with the results of this review. In research as well as in other areas, such as healthcare quality improvement, reviews have noted the special importance of creating equal relationships between patients and professionals in order to create successful patient engagement [61, 62]. The results of this review support that this is of prime importance.

Overall, the findings of this review provide insight into the patient-partners’ views of patient engagement, and the actions proposed in Table 3 are lines of thought to overcome the challenges of current patient engagement and to enhance its successes. It is hoped that by improving the understanding of patient-partners’ experiences, future implementations of patient engagement will be improved, which will lead to better experiences for patient-partners, more meaningful engagement, and patient-centered research of higher quality.

### Strengths and limitations

This systematic review has several strengths. For one, the high number of relevant articles identified, combined with the multiple contexts of patient engagement described in these articles, provided rich data. For example, studies including different populations of patient-partners engaging at different levels were identified, which allowed a great number of different perspectives to be included, yielding more generalizable findings. Furthermore, the methods of analysis, using line-by-line coding with inductive identification of themes to synthesize the most important concepts found in the literature, provided a detailed and thorough description of the experience of patient-partners in research. A variety of methods were used in the selected articles, including case studies, descriptive studies with semi-structured interviews, and broad questionnaire-based studies. This review benefited from this variety in methods, as it allowed to integrate different outlooks on the same data.

Some limitations were also identified. Since the vocabulary used to describe patient-partners is not standardized in the literature, the search strategy may have missed some relevant studies, and therefore concepts to analyse, even with the search strategy specifically designed to alleviate this issue. Other search strategies have been used to identify literature relating to patient engagement in research, such as the Medline filter published by Rogers et al*.* [63]. This validated search strategy was not used in this review. Nonetheless, the authors are confident in the search strategy used for this review, as it was developed carefully with the help of an information professional. Two main biases potentially affected the results: participation bias in larger descriptive studies and publication bias in case reports. For larger studies that described the experience of patient-partners from many projects, it is probable that more patient-partners who had positive experiences of research participated than those who had negative experiences. For case reports, successful implementations of patient engagement were probably more frequently reported than unsuccessful projects. As a consequence of these biases, this review may have failed to identify areas where patient-partners’ experience can be improved or overstated the positive aspects of patient engagement.

## Conclusions

Through the synthesis of qualitative studies on the experience of patient-partners in research, this systematic review provides a greater understanding of how patient engagement unfolds, as viewed by those most involved. Through seven themes detailing different aspects of this experience, this review provides insight into the strengths and weaknesses of current patient-engagement methods. It is hoped that through a better understanding of patient engagement, future research teams will be able to improve on current methods, which will ultimately lead to health research of better quality.

## Supplementary Information

**Additional file 1**. ENTREQ statement checklist” contains the in-text location of all recommended reporting elements of the ENTREQ statement.

**Additional file 2**. Search strategy and results” contains the complete search strategy used for each database, as well as the number of results found in each query.

**Additional file 3**. Quality assessment results” contains the complete results of the CASP qualitative checklist evaluation.

## Data Availability

The ReadCube Papers online library (Version 12.0) with search results and the NVivo file (NVivo 1.5.1) used for this study are available from the corresponding author on reasonable request.
